# Neuroenhancement by noninvasive brain stimulation is not a net zero-sum proposition

**DOI:** 10.3389/fnsys.2014.00127

**Published:** 2014-07-08

**Authors:** Bruce Luber

**Affiliations:** Division of Brain Stimulation, Departments of Psychiatry and Behavioral Sciences and Psychology and Neuroscience, Duke UniversityDurham, NC, USA

**Keywords:** TMS, zero-sum, cognitive enhancement, noninvasive brain stimulation, plasticity

Overview: Over the last decade, it has become increasingly clear that cognitive enhancement with noninvasive brain stimulation (NBS) is a real phenomenon. Recently, it has been suggested that such enhancements be viewed within the framework of a zero-sum game: that the performance enhancements found with NBS represent a re-allotment of finite processing resources, with the gains in one situation balanced by costs elsewhere. In examining the NBS literature, we have found that about half of reports of NBS enhancements may have been the result of resource reallocation, although it is not clear that a cost can be identified in each situation. Moreover, the other half of reports suggest that NBS can cause not a resource reallocation but an actual addition of resources available. It is suggested here that while it is important to examine whether costs occur with NBS, a more helpful framework from which to understand cognitive enhancements with NBS may be to understand brains as systems designed to continuously enhance their own functions and available resources (through learning, automatizing useful behaviors, etc.), and to view NBS as a means to augment these ongoing processes.

In recent years it has been increasingly apparent that cognitive enhancement via noninvasive brain stimulation (NBS), primarily using magnetic fields (with transcranial magnetic stimulation: TMS) and electric currents (e.g., transcranial direct current stimulation: tDCS), is a real phenomenon. Such enhancements are usually reported as increases in speed, and/or accuracy in the performance of various psychological tasks (McKinley et al., 2012; Luber and Lisanby, 2014). The mechanisms behind these increases in performance are still unclear. Recently, it was suggested that the mechanisms of enhancement could be thought of within a zero-sum framework (Brem et al., 2014).

This idea, which was said to be grounded on the physical principle of conservation of energy in closed systems, can be best expressed using the game theoretical concept of a zero sum game, where the sum of all gains amongst the players is zero: in certain games, if someone wins, someone else must have lost. Brem et al. extended this concept to neural systems: if the system is zero-sum, then gains (in the present case, those achieved via cognitive enhancement) must be balanced by costs (losses in function) somewhere else in the system. To the extent that processing in the brain can be considered zero-sum, there are direct and important implications to any program using NBS- primarily, when considering cognitive enhancement, one should look not only for the gains, but also the losses, and judge the whole with a cost/benefit analysis.

From a practical and ethical point of view, Brem et al. (2014) raised an important issue: whether, in their search for performance enhancement, researchers may not be paying enough attention to potential adverse side effects of NBS, a point recently made by others as well (Davis et al., 2013). However, Brem et al. (2014) was presented as a theoretical framework for interpreting cognitive enhancement effects produced using NBS. In this regard, it should first be mentioned that in no literal sense can the brain be considered a closed system as defined within thermodynamics, so whatever “grounding” was intended by Brem et al. (2014) is at best an analogy. However, a game theoretical approach might be applicable in the context of central executive functions. Many psychological processes exhibit limited capacity. The iconic examples are selective attention (going back to the cocktail party effect: Cherry, 1953) and working memory (e.g., the magic number 7 ± 2: Miller, 1956). Human information processing theories in the 1960s formulated such processing using a computer analogy, with the central executive being like a CPU. In the 1970s, models using resource theories (e.g., Kahneman, 1973), some borrowed from economics (e.g., Navon and Gopher, 1979) were also used to explain limited capacity phenomena. The gist of such modeling is to assume we have a limited set of processing resources under control of a central executive processor which attempts to deploy these resources in an optimal manner to maximize performance. The limited amount of computational capacity to achieve this deployment of resources can be conceptualized as processing power. Differential allotments in resources deployed can be observed in such phenomena as the speed-accuracy trade-off (SATO), where higher accuracy can be achieved by sacrificing greater speed and vice-versa, or in changes in detection accuracy of visual targets depending on how spatial attention is divided or focused in the visual field.

What might be the consequences of NBS enhancement of performance on such a system having a limited capacity central processor deploying a finite set of resources (say for working memory or attention)? In one report of NBS enhancement, subjects were to detect small rectangles appearing to the left or right visual field, and 1 Hz rTMS (which is thought to temporarily down-regulate cortical excitability, e.g., Chen et al., 1997) increased detection accuracy for ipsilateral stimulation- but at a cost of lower accuracy for items appearing in the contralateral field (Hilgetag et al., 2001). In another experiment, participants were to search an array of objects for a target, defined by some combination of form, color, and motion features (Walsh et al., 1998). When V5, a posterior cortical area central to motion processing, was stimulated with TMS while the visual search array was presented (disrupting motion processing), subjects had enhanced reaction times when the target did not include motion as an essential feature- but at a cost of slowing performance when it did. These two studies demonstrate that one can create enhanced performance with TMS but this can come at a cost for other types of performance.

In a recent review of cognitive enhancement through TMS (Luber and Lisanby, 2014), we termed this sort of phenomenon “addition-by-subtraction,” and found 26 instances of it in 62 studies reporting TMS enhancement effects (a more generalized discussion of “enhancement through diminishment” can be found in Earp et al., 2014). We suggested that the addition-by-subtraction mechanism appears to function by disrupting or inhibiting an inessential or less essential but competing part of one or more functional brain networks involved in a task, resulting in temporary network reorganization. This sort of explanation seems in agreement with the zero-sum framework of Brem et al. (2014). However, in many cases of addition-by-subtraction we enumerated it is difficult to identify a cost. For example, in one visual search task, the items in the search array look like tilted “x”s, with the difference between target and distractors being the direction of the tilt. There is a natural learned tendency to identify the items in the array as “x”s and to mentally correct their tilt to the canonical orientation of an x, which makes the search for the item with opposite tilt less efficient. After many trials, individuals learn to overcome this tendency and search more efficiently. On the other hand, subjects who received 1 Hz rTMS to parietal cortex before starting this visual search task are immediately speeded in their performance, presumably because the NBS inhibited the overlearned tendency (Oliveri et al., 2010). Temporarily inhibiting this process so that learning can take place more quickly hardly seems like a cost. On the contrary, inhibiting counterproductive tendencies or processes to make learning more efficient appears to be a promising use of NBS. While NBS did cause a change in how neural resources were used, it is not clear that there was a cost in Oliveri et al. (2010), since what was being temporarily inhibited was a tendency counterproductive to learning.

As presented by Brem et al. (2014), within a zero-sum conceptualization enhancement effects caused by NBS primarily occur via direct or indirect alterations in allotment of neural resources. While close to half of the reports of NBS-caused performance enhancements we found appeared to be of this sort (although not all of those entailed what most would deem a “cost”), more than half did not fit such a framework (Luber and Lisanby, 2014). In these studies, NBS enhanced performance with direct application to a cortical region necessary to processing of the task. Crucially, the best explanation for these enhancements was not that NBS caused greater allotment of neural resources to this region and a loss of resources elsewhere, but that the stimulation in some way added to the resources available to process the task. An example of this is the proposed mechanism of stochastic resonance, in which lower intensities of NBS add enough to neural activity to push it over threshold for detection in some instances, enhancing perceptual sensitivity (Miniussi et al., 2010).

The zero-sum conception thus makes sense when the NBS is causing a change in a limited capacity system to increase processing resources in one part of the system to the detriment of another part. It does not address a situation in which the NBS is actually increasing overall resources and capacity. One useful framework to make the distinction involves diffusion models of perceptual decisions, where there are two primary ways to speed decisions. First, one could lower the decision criterion, which does create costs and benefits: a speed-accuracy trade-off. On the other hand, the size of the individual steps made in the random walk toward the decision boundaries could be made larger by increasing the efficiency of the processing. This increase in processing resources speeds reaction time, but does not entail a cost. This latter mechanism may explain the results when 5 Hz rTMS was applied to lateral occipital complex in sleep deprived subjects, dramatically enhancing reaction time without incurring a cost in accuracy (Luber et al., 2008).

Beyond acute changes caused by NBS, which can in some cases act to increase innate capacity, the great promise for cognitive enhancement lies in more permanent improvements, whether in remediating deficits in neurological or psychiatric patients or in enhancing the skills of healthy individuals. Especially in the latter case, some rudimentary steps have been made toward developing a technology that uses NBS to augment learning. Two manipulations appear to be of importance to creating long-lasting cognitive enhancement: first, repeated NBS sessions to generate a cumulative effect (Thut and Pascual-Leone, 2009), and second, stimulating task-relevant cortical regions while concurrently activating them with task performance, creating Hebbian synergies in neurons directly related to performance (Thickbroom, 2007). For example, four 1-h sessions of concurrent TMS/working memory task performance over the course of 2 days of sleep deprivation resulted in complete remediation of sleep deprivation deficits in working memory, while subjects receiving sham TMS had the normal lapsing and reaction time slowing caused by lack of sleep. This effect lasted at least 18 h beyond the last TMS session (Luber et al., 2013). Research in the NBS field is only struggling through crude beginnings, but as we learn to integrate the optimal pulse waveforms at the optimal sets of frequencies for the right durations and places at the best intensities, timed with the appropriate cognitive tasks, we may learn to dramatically accelerate the learning of desired skills.

It is in this sense that using a zero-sum framework in the context of NBS cognitive enhancement is not particularly appropriate. Yes, it is important to remember that at any one point in time the capacities of the brain are finite, just as it is essential to understand that the order-generating processes of living organisms occur against the background of the increasing entropic gradient imposed by the second law of thermodynamics. However, a key element of nervous system organization is the way it adapts, remembers and learns at all levels of organization from synapse to cell assembly to systems of nuclei and cortical regions, over the course of seconds, minutes, weeks, and ages. The nervous system is designed to keep redesigning itself, to keep enhancing its capabilities. As one example, much of the brain is organized to continuously automatize its behaviors, to free up the limited-capacity executive processing system designed to deal with novel situations. What before took all of that executive system to deal with later does not occupy it at all as skills are learned (think the first time you tried to drive a car and how effortless it is now). Through such mechanisms as automatization, the brain in essence constantly expands its available resources, despite having a finite processing capacity and resource repertoire at any given instant. Because of this, perhaps the most appropriate framework to develop NBS cognitive enhancement is within a conceptualization of the human brain as the enhancement system it already is. Using NBS to enhance cognition is just another way our brains have found to operate on and improve themselves, this time by using what they are learning about themselves to enhance their operation by direct stimulation from without.

## Conflict of interest statement

The author declares that the research was conducted in the absence of any commercial or financial relationships that could be construed as a potential conflict of interest.
